# Structurally characterized terminal manganese(iv) oxo tris(alkoxide) complex†
†Electronic supplementary information (ESI) available: Single crystal X-ray diffraction data; ESI-MS spectra and data: IR spectra; and magnetic data. CCDC 1829119–1829121. For ESI and crystallographic data in CIF or other electronic format see DOI: 10.1039/c8sc01164h


**DOI:** 10.1039/c8sc01164h

**Published:** 2018-04-26

**Authors:** Robert L. Halbach, David Gygi, Eric D. Bloch, Bryce L. Anderson, Daniel G. Nocera

**Affiliations:** a Department of Chemistry and Chemical Biology , Harvard University , Cambridge , Massachusetts 02138 , USA . Email: dnocera@fas.harvard.edu

## Abstract

A Mn(iv) complex featuring a terminal oxo ligand, [Mn^IV^(O)(ditox)_3_][K(15-C-5)_2_] (**3**; ditox = ^*t*^Bu_2_MeCO^–^, 15-C-5 = 15-crown-5-ether) has been isolated and structurally characterized. Treatment of the colorless precursor [Mn^II^(ditox)_3_][K(15-C-5)_2_] (**2**) with iodosobenzene affords **3** as a green free-flowing powder in high yields. The X-ray crystal structure of **3** reveals a pseudotetrahedral geometry about the central Mn, which features a terminal oxo (*d*(Mn–O_term_ = 1.628(2) Å)). EPR spectroscopy, SQUID magnetometry, and Evans method magnetic susceptibility indicate that **3** consists of a high-spin *S* = 3/2 Mn(iv) metal center. **3** promotes C–H bond activation by a hydrogen atom abstraction. The [Mn^IV^(O)(ditox)_3_]^–^ furnishes a model for the proposed terminal oxo of the unique manganese of the oxygen evolving complex of photosystem II.

## Abstract 2

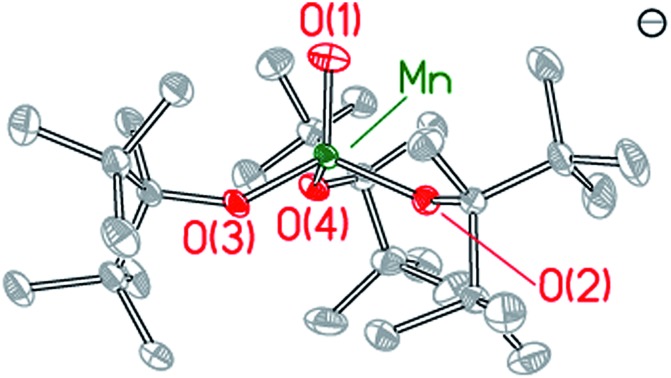
First structurally characterized Mn(iv) oxo.

High valent metal oxos are a linchpin for manipulating the O–O bond in energy conversion transformations involving the oxygen reduction reaction (ORR) and its reverse process, the oxygen evolution reaction (OER).^1^,^2^ For metals left of the oxo wall,^3^ a terminal metal oxo is often invoked as the primary product of O–O bond cleavage of the ORR, and conversely, O–O bond formation of the OER is often proposed to proceed from a high-valent metal oxo. In the OER conversion of photosystem II, the “dangler” manganese,^4^ of the manganese oxygen evolving complex (OEC) is proposed to support a terminal metal oxo in the high valent S4 state of the Kok cycle.^5^–^12^ Most high valent manganese oxos are proposed as transient intermediates in oxidation reactions.^13^–^16^ In this regard, the stabilization of the terminal Mn-oxo moiety by hydrogen bonding within the trigonal ligand field of [buea]^3–^ (tris[(*N*′-*tert*-butylureaylato)-*N*-ethylene]aminato) is notable for its elegant and concise design^17^,^18^ as is the stabilization of a terminal oxo in tetragonal ligand fields of nitrogen-donating macrocycles.^19^ Nonetheless, in any ligand field, isolation and structural characterization of high valent Mn oxos remain elusive with the peculiarity that oxos in their higher formal oxidation state of Mn(v) are better structurally characterized^19^–^22^ than their Mn(iv) counterparts.

The reactivity of the metal-oxo functional group is largely governed by the coordination geometry about the metal center. The preponderance of metal-oxo complexes, including that for Mn,^19^–^23^ feature a pseudo-octahedral or tetragonal ligand field,^24^ beginning with the early description of the electronic structure of the vanadyl oxo.^25^ When metals have higher d electron counts, the oxo is better stabilized in the more uncommon trigonal ligand field,^26^,^27^ as demonstrated by the C_3_ symmetry of the [H_3_buea]^3–^ ligand.^28^ We have explored the exceptionally weak trigonal ligand field engendered by the tris(alkoxide) platform of ditox (Hditox = ^*t*^Bu_2_MeCOH)^29^ to support the metal-oxo unit.^30^,^31^ The steric bulk of the ditox ligand is less than that of the related tritox^32^,^33^ ligand as a result of the replacement of one *tert*-butyl group for one methyl group. The ditox ligand allows for the preferential formation of tris(alkoxide) metal complexes, which readily accommodate a terminal-oxo in a pseudotetrahedral ligand field upon oxygen-atom transfer, as has been demonstrated for a series of 3d transition metals (M = V, Cr, Fe).^30^,^31^ Herein, we show that a trigonal ligand field of the ditox ligand can support a Mn(iv) terminal oxo. The isolation, structural characterization and reactivity of [Mn(O)(ditox)_3_][K(15-C-5)_2_] (**3**) provides a benchmark for Mn(iv) in an exclusive oxygen ligand field, as is the case for the Mn(iv) centers of the oxygen evolving complex (OEC) of photosystem II.

The preparative reaction chemistry utilized to obtain Mn(ii) and Mn(iv)-oxo compounds in the tris(ditox) environment is outlined in Fig. 1. The addition of three equivalents of K(ditox) to MnCl_2_ in THF at ambient temperature readily furnishes a colorless solid and a faint yellow solution of Mn^II^(ditox)_3_K(THF)_2_ (**1**). Filtration, removal of solvent *in vacuo*, and crystallization from pentane at –40 °C gives analytically pure colorless crystals of **1** in 54% yield. The potassium salt, [Mn^II^(ditox)_3_][K(15-C-5)_2_] (**2**), is prepared by treatment of a pentane solution of **1** with two equivalents of 15-C-5 at room temperature, quantitatively furnishing **2** as a colorless fine powder. Crystals of **2** suitable for X-ray diffraction can be obtained from a concentrated Et_2_O solution cooled at –40 °C overnight. Treating **2** with one equivalent of PhIO in THF at room temperature rapidly generates a dark green solution of [Mn^IV^(O)(ditox)_3_][K(15-C-5)_2_] (**3**). Filtration, removal of solvent *in vacuo*, and multiple pentane washings of the resultant solid furnishes **3** as an intensely dark green powder in 79% yield following drying *in vacuo*. Solutions of **3** stored under an inert atmosphere at ambient temperature change from dark green to brown over the course of a few hours, indicating the decomposition of **3**, which can be stored indefinitely as a solid in the dark at –40 °C. The negative mode ESI-MS spectrum of **3** (Fig. S1†) exhibits a prominent ion peak at *m*/*z* 542.4 with mass and isotopic distribution patterns corresponding to [Mn^IV^(O)(diox)_3_]^–^ (calcd *m*/*z* 542.4). A mass shift from *m*/*z* 542.4 to 544.4 was observed when **3** was generated using ^18^O-enriched PhIO (46% ^16^O: 54% ^18^O). Crystals of **3** suitable for X-ray diffraction can be obtained from a concentrated Et_2_O solution of **3** cooled overnight at –40 °C.

**Fig. 1:**
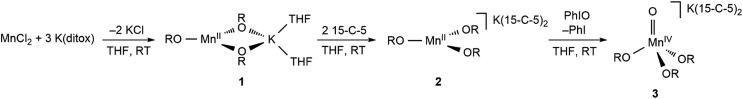
Preparative reactions of Mn ditox compounds, where OR = ditox and 15-C-5 = 15-crown-5-ether.

Single crystal X-ray diffraction studies were performed on compounds **1–3**. Details of the data collection are provided in Table S1† and X-ray crystal structures are shown in Fig. 2 and in Fig. S2–S4,† which also provide selected bond lengths and angles for the complexes. Compound **1** crystallizes in the monoclinic space group *C*2*c* and exhibits a Y-shaped geometry about manganese with the potassium cation coordinated directly to two ditox ligands (Fig. 2). The average Mn–O_alk_ bond distance is 1.941 ± 0.052 Å. The sum of the O_alk_–Mn–O_alk_ bond angles of **1** is 359.2°, highlighting the planar nature of the compound and indicating that the manganese center resides in the plane of the alkoxides. However, the O(1)–Mn(1)–O(1A) bond angle of 97.64(8)° in **1** is significantly contracted relative to the two other O–Mn–O angles in the trigonal plane. One ditox ligand of **1** was disordered over two positions in a 53 : 47 ratio, and each THF molecule was disordered over two positions in a 50 : 50 ratio.

**Fig. 2:**
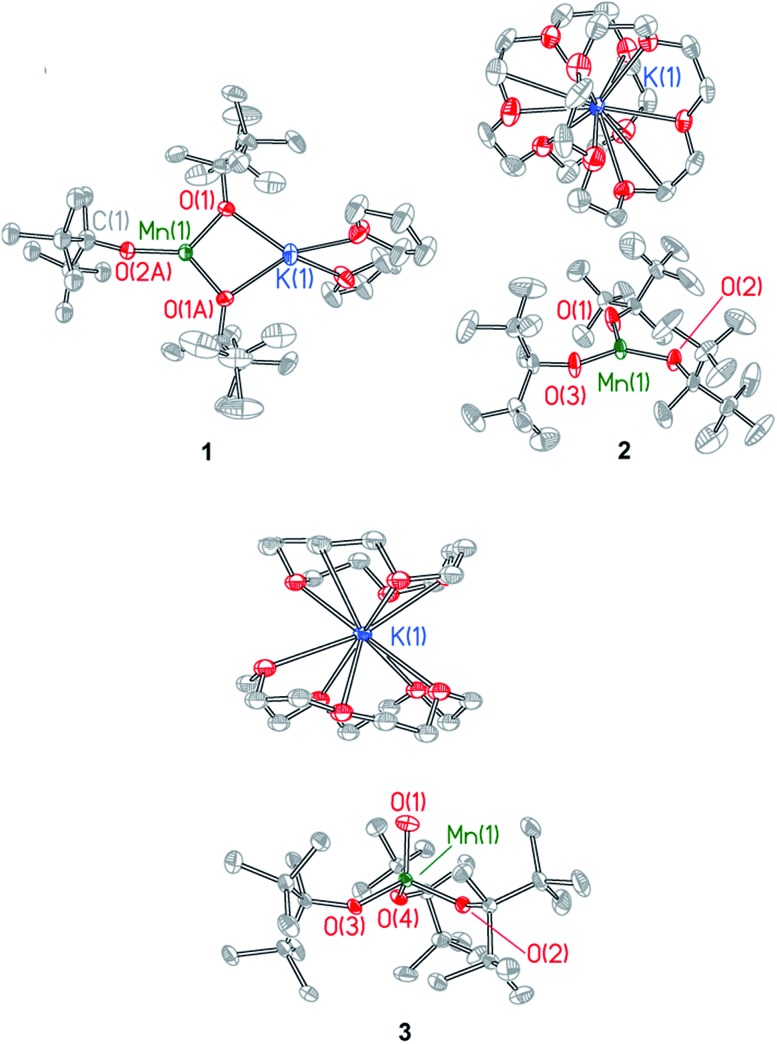
X-ray structures of Mn(ii) complexes **1** and **2** and Mn(iv)-oxo complex **3**. Selected bond distances and angles are provided in the ESI.†

Compound **2** crystallizes in the monoclinic space group *P*2_1_/*c* as a discrete anion/cation pair (Fig. 2). The average Mn–O_alk_ bond distance is 1.925 ± 0.003 Å, and the sum of the O_alk_–Mn–O_alk_ bond angles is 359.8°, indicating the trigonal planar nature of **2**. The sequestration of the potassium countercation by two crown ether molecules prevents distortion of the O_alk_–Mn–O_alk_ bond angles and renders a pseudo-*D*_3h_ symmetry about manganese in which each angle is very nearly 120°.

Compound **3** crystallizes in the triclinic space group *P*1 as a discrete anion/cation pair (Fig. 2). The manganese center exhibits a slightly axially distorted pseudotetrahedral geometry with approximate *C*_3v_ symmetry. The terminal Mn–O_term_ function has a bond length of 1.628(2) Å and is bent at an angle of 85.86(8)° relative to the plane defined by the alkoxide oxygen atoms. The average Mn–O_alk_ bond distance is 1.849 ± 0.024 Å, which is notably shorter than the average Mn–O_alk_ bond distance of **1** and **2**, consistent with the higher oxidation state of manganese in **3**. The average O_term_–Mn–O_alk_ bond angle is observed to be 111.23 ± 4.52° while the average O_alk_–Mn–O_alk_ bond angle is 107.71 ± 3.69°, an angle that is consistent with a pseudotetrahedral geometry. One of the crown ether molecules is disordered over two positions in a 50 : 50 ratio.

The nature of the Mn(iv)–O_term_ is in line with a wide range of metal oxo complexes. Table 1 lists structural metrics for a series of related compounds to **3**. Beginning with a trigonal ditox ligand platform, the Mn–O_term_ bond distance in **3** is similar to its V and Cr counterparts of d^0^ and d^1^ electron counts. Inasmuch as the d^0^–d^4^ systems occupy the same e-orbital set of primarily d_*xz*/*yz*_ character, the similarity of the d(Mn–O_term_) bond distances for M = V, Cr and Mn is unsurprising. The d(Mn–O_term_) bond distance, which is much shorter than that of hydroxides bound to metals in a ditox trigonal field as reflected by Fe(iii)(OH)(ditox)_3_^–^, lies between the Mn(iv)-oxygen single bond distance and the Mn(v)-oxygen triple bond distance of structurally characterized manganese complexes with a terminal oxygen ligand (Table 1). Additionally, though not crystallographically characterized, Mn(iv)=O bond lengths have been determined by EXAFS for two Mn(iv) oxo compounds (see Table 1), and they are in line with that observed for **3**.

**Table 1:** Selected terminal metal–oxygen bond lengths

M–O moiety	Complex	*d*(M–O)/Å	Reference
Mn(iv)–O_term_	MnO(ditox)_3_^–^	1.628(2)	This paper
Mn(iv)–O_term_	MnO(BnTPEN)^2+^^*g*^	1.69^*b*^	43
Mn(iv)–O_term_	MnO(OH)(Me_2_EBC)^2+^^*h*^	1.71^*b*^	34
Cr(v)–O_term_	CrO(ditox)_3_	1.649(2)	31
V(v)–O_term_	VO(ditox)_3_	1.605(1)	31
Fe(iii)–OH_term_	Fe(iii)(OH)(ditox)_3_^–^	1.890(1)	30
Mn(iv)–OH_term_	Mn(iv)(OH)(buea)^–^^*a*^	1.83^*b*^	35
Mn(v)–O_term_	MnO(TAML)^–^^*c*^	1.549(3)	20
Mn(v)–O_term_	MnO(TAML′)^–^^*d*^	1.5555(12)	19
Mn(v)–O_term_	MnO(PHAB)^–^^*e*^	1.558(4)	21
Mn(v)–O_term_	MnO(TBP_8_Cz)^*f*^	1.5455(18)	22

^*a*^[H_3_buea] = tris[(*N*′-*tert*-butylureaylato)-*N*-ethylene]amide.

^*b*^Distance determined from EXAFS.

^*c*^H_4_TAML = 6,6-diethyl-3,3,9, tetramethyl-1,8,9,11-tetrahydro-4λ2-pyrido[2,3-*e*][1,4,7,10]tetraazacyclo-tridecine-2,5,7,10(3*H*,6*H*)-tetraone.

^*d*^H_4_TAML′ = 3,4,8,9-tetrahydro3,3,6,6,9,9-hexamethyl-1*H*-1,4,8,11-benzotetraazocyclotridecane-2,5,7,10(6*H*,11*H*)tetrone.

^*e*^H_4_PHAB = 1,2-bis(2,2-diphenyl-2-hydroxyethanamido)benzene.

^*f*^H_3_TBP8Cz = octakis(*p-tert*-butylphenyl)corrolazine.

^*g*^BnTPEN = *N*-benzyl-*N*,*N*′,*N*′-tris(2-pyridylmethyl)-1,2-diaminoethane.

^*h*^Me_2_EBC = 4,11-dimethyl-1,4,8,11-tetraazabicyclo[6.6.2]hexadecane.

The FTIR spectra of **1–3** are reproduced in Fig. S5.† The M–OR stretching region of **1** shows a strong absorption at 575 cm^–1^ as well as two weak absorptions at 637 cm^–1^ and 659 cm^–1^. The addition of 15-C-5 to **1** to furnish **2** results in the lowest-energy band of **1** shifting from 575 cm^–1^ to 583 cm^–1^ and the two higher-energy bands of **1** coalescing into a single band at 646 cm^–1^. The expansion of one of the O_alk_–Mn–O_alk_ bond angles of **2** relative to **1** appears to be correlated with a higher-energy M–OR feature in **2**. The lower-energy band of **2** shifts from 583 cm^–1^ to 591 cm^–1^ and the higher-energy band shifts from 646 cm^–1^ to 649 cm^–1^ upon its reaction with PhIO to give **3**. These shifts to higher energy are consistent with the higher oxidation state of **3** and the attendant decrease in the Mn–O_alk_ bond distance. The FTIR spectra of **3** and its ^18^O_oxo_-isotopomer (Fig. S6†) display Mn–O_oxo_ stretching frequencies at 845 cm^–1^ and 809 cm^–1^ (*ν*(Mn^16^O)/*ν*(Mn^18^O) = 1.04; calcd 1.06), respectively.

Solution susceptibility measurements of **1** and **2** at 298 K yield *μ*_eff_ values of 6.03 *μ*_B_ and 5.77 *μ*_B_, respectively, which is consistent with both complexes possessing a high-spin *S* = 5/2 spin state (*μ*_spin-only_ = 5.92 *μ*_B_). The solution susceptibility measurement of **3** at 298 K yields a *μ*_eff_ value of 4.54 *μ*_B_, which is comparable to values for other observed Mn(iv)–oxo complexes possessing a spin state of *S* = 3/2 (*μ*_spin-only_ = 3.87 *μ*_B_).^23^,^36^ SQUID DC magnetic susceptibility of powdered **3** from 2–298 K confirms a high-spin configuration, which is consistent with the weak-field nature of the ditox ligand.^29^ SQUID magnetometry at 20 °C provides a solid-state susceptibility of 4.75 *μ*_B_ for **3**, which is significantly greater than the spin-only susceptibility of 3.87 *μ*_B_ expected for an isotropic *S* = 3/2 system (Fig. S7†) and is more in-line with a highly anisotropic species. Indeed, X-band EPR spectroscopy performed in perpendicular mode on **3** shows a pseudo-axial signal at *g*_*x*_ = 4.135, *g*_*y*_ = 3.860, and *g*_*z*_ = 1.980 (Fig. 3). Sharp hyperfine coupling can be observed at *g*_*z*_ yielding |*A*_*z*_| = 299 MHz. A plot of *χT versus T* for **3** shows antiferromagnetic behavior with *χT* decreasing steadily as temperature decreases to ∼20 K, after which *χT* drops quickly to a minimal value of 0.35 cm^3^ K mol^–1^ at 2 K (Fig. S7†). The large temperature dependence of *χT* suggests the presence of significant magnetic anisotropy, as anisotropic spins possess energy separations between their *M*_S_ levels that are often within an order of magnitude of *k*_B_T, leading to temperature dependent behavior. Attempts to model the *χT versus T* data without considering magnetic anisotropy were unsuccessful. In order to attempt to quantify the axial zero-field splitting parameter *D*, the *χT versus T* data (Fig. S8†) were fit to the Hamiltonian: *Ĥ* = *Dŝ*_*z*_^2^ + *μ*_B_*g****S***·***H***, where *D* is the zero-field splitting, *ŝ*_*z*_ is the spin operator, *μ*_B_ is the Bohr magneton, *g* is the Landé *g*-factor, **S** is the spin, and **H** is the magnetic field. The model employs only axial *D* and *g*-tensors, ignoring any transverse anisotropy (*E*) since including this term did not improve the fit to the data and resulted in *g*-factors that varied significantly from those determined *via* EPR spectroscopy. The best fit to the data produced *g*_*x*_, *g*_*y*_, and *g*_*z*_ values of 4.15, 3.57, and 1.92, respectively, and a value of *D* of –12.31 cm^–1^, all of which are in good agreement with the values obtained from EPR. To more accurately determine the magnitude of *D* and *E*, we collected low-temperature magnetization data at various applied DC fields. The resulting plot of reduced magnetization for **3** is shown in Fig. S8.† To quantify the effect of strong magnetic anisotropy, the data were modeled according to the Hamiltonian: *Ĥ* = *Dŝ*_*z*_^2^ + *E*(*ŝ*_*x*_^2^ – *ŝ*_*y*_^2^) + *μ*_B_*g****S***·***H*** which is similar to the above mentioned model but contains an additional transverse zero-field splitting parameter, *E*. Fits to the data using PHI^37^ and an average *g*-factor of 3.13 give axial and transverse zero-field splitting parameters of *D* = –23.4 cm^–1^ and |*E*| = 6. This *D*-value is several orders of magnitude larger than those reported for a suite Mn(iv)(OH) compounds.^38^,^39^

**Fig. 3:**
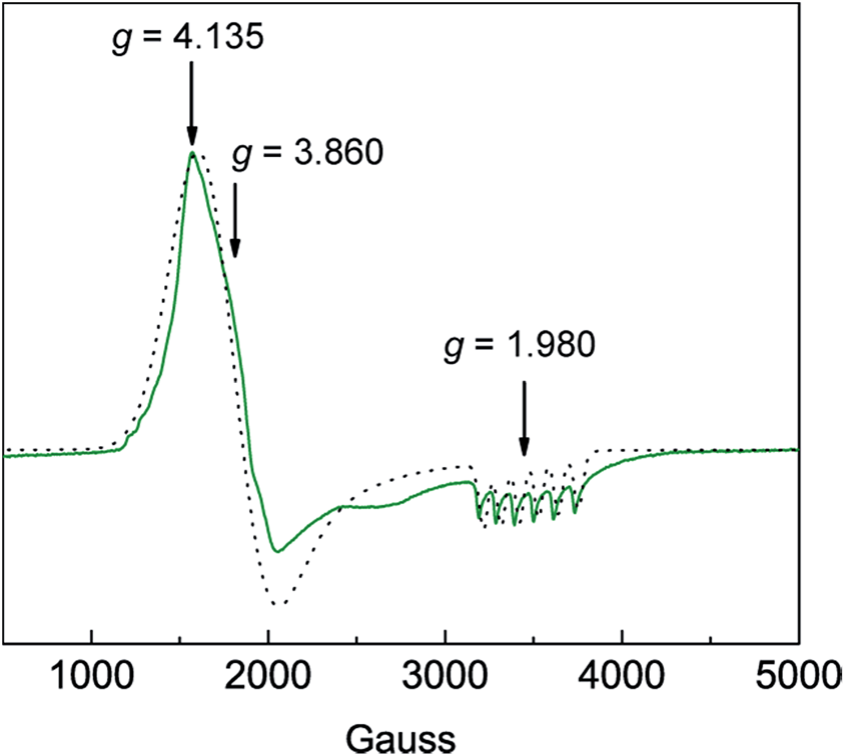
Experimental (green line 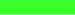) and simulated (black dots 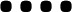) X-band CW-EPR spectrum of [Mn^IV^(O)(ditox)_3_][K(15-C-5)_2_] (**3**) at 6 K in butyronitrile glass. See text for simulation parameters.

Compound **3** can be oxidized under mild conditions. Cyclic voltammograms of **3** in a 0.2 M [TBA][PF_6_]/THF solution show an electrochemically reversible oxidation wave with an *E*_1/2_ value of –0.65 V *versus* Fc^+/0^ that we attribute to the Mn(v/iv) couple (Fig. 4). A cathodic sweep to negative potentials engenders an irreversible reduction wave with a peak current at –2.27 V *versus* Fc^+/0^. This irreversible reduction wave is accompanied by the visible formation of a brown insoluble solid.

**Fig. 4:**
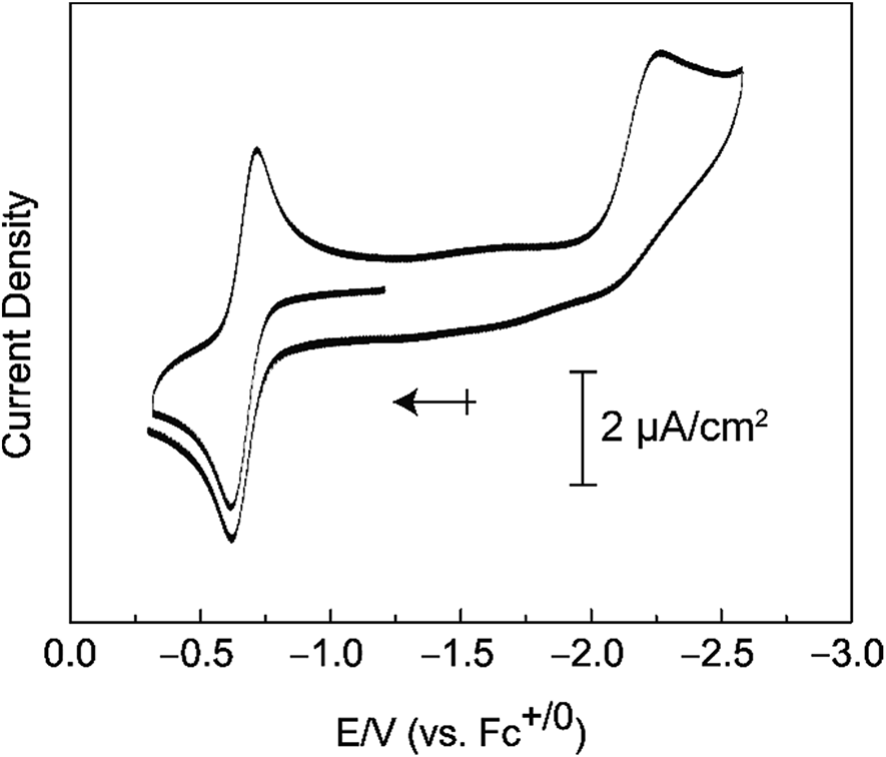
Cyclic voltammogram of [Mn^IV^(O)(ditox)_3_][K(15-C-5)_2_] (**3**) in THF/[TBA][PF_6_].

Complex **3** reacts with the relatively weak C–H bonds of 9,10-dihydroanthracene (76.2 kcal mol^–1^)^40^ and 1,4-cyclohexadiene (CHD) (76.0 kcal mol^–1^)^40^ and produces the Mn(iii) hydroxide, as the properties of the product in color and solubility are similar with the previously reported Fe^III^–OH ditox compound.^30^ The reaction between **3** and excess CHD generates nearly half of an equivalent of benzene and trace amounts of 1,3-cyclohexadiene (Fig. S10†). Attempts to oxidize stronger C–H bonds, such as those of ethyl benzene (85.4 kcal mol^–1^),^40^ show no reaction by ^1^H NMR. Notably **3** also does not react with oxygen-atom acceptors. For instance, treatment of **3** with a ten-fold excess of PPh_3_ shows no evidence of the oxygen-atom transfer product, OPPh_3_, or any other phosphorous-containing products, as indicated by ^31^P{1H} NMR. This lack of oxygen-atom transfer reactivity is similar to that observed for the nucleophilic Cr d^1^ complex [CoCp_2_][O_2_Cr(NRAr)_2_] prepared by Cummins and coworkers,^41^ who contrast the reactivity of their nucleophilic Cr d^1^ complex against that of the electrophilic Re d^1^ complex [HB(pz)_3_]ReO_2_Cl prepared by Mayer and coworkers^42^ who concluded that the highly electrophilic nature of the oxidized complex [HB(pz)_3_]ReO_2_Cl^+^ was mainly a result of its cationic charge. In turn, Cummins and coworkers attribute the lack of oxidizing ability of their complex to its negative charge in conjunction with the strongly electron-donating amido ligands. Similarly, we believe that the lack of oxygen atom transfer chemistry of **3** is the result of its anionic charge and strong electron-donating properties of the ditox ligand set. For example, in a neutral all N-donor field, a similar manganese oxo prepared by the group of Nam is able to attack the much stronger bonds of cyclohexane (99.5 kcal mol^–1^).^43^

The ability of the all oxygen anionic ligand field of ditox to stabilize a Mn(iv) oxo may shed light on the ability of the Oxygen Evolving Complex (OEC) to protect itself in the S3 state proposed by Lubitz in which all manganese centers are in the +4 oxidation state.^6^ As for [Mn^IV^(O)(ditox)_3_]^–^, the OEC has an anionic all oxygen ligand field, suggesting that a muted reactivity of the Mn(iv)-oxo in a donating oxygen ligand field may be a strategy for the OEC to circumvent the oxidation of the amino acid environment in lower S states of the Kok cycle such as S3, thus preserving the OEC to perform water splitting in the higher and more reactive S4 oxidation state. In this regard, the Mn(iv) oxo ditox system suggests a mechanism by which the reactivity of traditionally high energy reactive intermediates may be attenuated in a pre-catalytic resting state, a property that protein cofactors such as OEC have long mastered.

## Conflicts of interest

There are no conflicts to declare.

## Supplementary Material

Supplementary information

Crystal structure data
