# Supplementary material for: Structurally characterized terminal manganese(iv) oxo tris(alkoxide) complex
Source: Chem Sci. 2018 Apr 26;9(19):4524–8. doi: 10.1039/c8sc01164h (PMC5958342; doi:10.1039/c8sc01164h)
Supplement: Supplementary file 1 [file SC-009-C8SC01164H-s001.pdf]

Electronic Supplementary Material (ESI) for Chemical Science.
This journal is © The Royal Society of Chemistry 2018

Supporting Information

# Structurally Characterized Terminal Manganese(IV)-Oxo Tris(alkoxide) Complex

Robert L. Halbach, David Gygi, Eric D. Bloch, Bryce L. Anderson and Daniel G. Nocera*

*Department of Chemistry and Chemical Biology, Harvard University, 12 Oxford Street, Cambridge, MA 02138–2902*

Email: dnocera@fas.harvard.edu

| *Index* | *Page* |
|---|---|
| General methods | S2–S3 |
| X-ray crystallographic details | S4 |
| SQUID magnetometry details | S4 |
| Syntheses and characterization of compounds | S5–S6 |
| ESI-MS spectrum of compounds **3** and $^{18}O_{oxo}$-enriched **3** | S7 |
| X-ray crystallographic table | S8 |
| X-ray structures of compounds **1**, **2**, and **3** | S9–S11 |
| Infrared spectra of compounds **1**, **2**, and **3** | S12–S13 |
| SQUID magnetization data for **3** | S14–S15 |
| UV-vis absorption spectra of compound **3** | S16 |
| NMR spectrum of reaction of **3** with cyclohexadiene | S17 |

## General methods

Reactions involving air-sensitive materials were performed in a nitrogen-filled glovebox. Glassware and magnetic teflon stirbars were dried at 150 °C for at least 12 h prior to use. Pentane was dried and distilled over sodium and stored over 4-Å molecular sieves. Diethylether ($Et_2O$), tetrahydrofuran (THF) and tetrahydrofuran-$d_8$ (THF-$d_8$) were dried and distilled over sodium and benzophenone and stored over 4-Å molecular sieves for at least 12 h. Butyronitrile was distilled from $P_2O_5$ and stored over 4-Å molecular sieves for several days prior to use. The reagents 15-crown-5-ether (15-C-5) and 1,4-cyclohexadiene (CHD) were rigorously freeze-pump-thawed under high-vacuum ($10^{-7}$ mTorr) several times and stored over activated 4-Å molecular sieves for several days prior to use. The reagent 9,10-dihydroanthracene (DHA) was recrystallized three times from THF solutions at –40 °C. ${}^{t}Bu_2MeCOH$ and ${}^{t}Bu_2MeCOK$ (Hditox and Kditox, respectively) were prepared according to literature methods.[1,2] A large scale preparation method is provided as previous reports do not explain how to work up the reaction. Butyronitrile, 15-C-5, CHD, DHA, ferrocene ($Fc^0$), methyllithium solution (1.6 M in diethyl ether), manganese dichloride ($MnCl_2$), pentane, $D_3PO_4$, tetrabutylammonium hexafluorophosphate ([TBA][$PF_6$]), THF, 2,2,4,4-tetramethyl-3-pentanone, and $PPh_3$ were obtained from Sigma-Aldrich. THF-$d_8$ was obtained from Cambridge Isotope. $^{18}O$-enriched iodosobenzene ($PhI^{18}O$, ca. 50% labeled) was prepared according to literature procedures.[3] Triphenylphosphine ($PPh_3$) was used as received.

Reaction products were characterized by IR, NMR and elemental analyses. Elemental analyses were performed by Complete Analysis Laboratories (Parsippany, NJ). IR spectra of crystalline samples were recorded on a PerkinElmer Spectrum 400 FT-IR/FT-FIR Spectrometer outfitted with a Pike Technologies GladiATR attenuated total reflectance accessory with a monolithic diamond crystal stage and pressure clamp. Samples were suspended in Paratone-N oil, and a background IR spectrum of Paratone-N oil was collected prior to data acquisition. $^{1}H$ NMR spectroscopy was performed on Varian Mercury 400B and Varian Unity/Inova 500/500B/500C NMR spectrometers with chemical shifts ($\delta$/ppm) referenced to residual proteo-solvent signals. $^{31}P\{^{1}H\}$ NMR spectroscopy was performed on a Mercury400B spectrometer with chemical shifts ($\delta$/ppm) referenced to phosphoric acid-$d_3$ solution ($D_3PO_4$; 85 wt. % in $D_2O$).

Solution magnetic moments were determined in THF at ambient temperature on a Varian Mercury400B NMR spectrometer using Evans method.[4] Pascal constants were used to estimate diamagnetic corrections.[5]

---

[1] Napolitano, E.; Giovani, E.; Ceccarelli, N.; Pelosi, P. *J. Agric. Food Chem.* **1996**, *44*, 2806–2809.

[2] Chambers, M. B.; Groysman, S.; Villagrán, D.; Nocera, D. G. *Inorg. Chem.* **2013**, *52*, 3159–3169.

[3] Schardt, B. C.; Hill, C. L. *Inorg. Chem.* **1983**, *22*, 1563–1565.

[4] Evans, D. F. *J. Chem. Soc.* **1959**, 2003–2005.

[5] Bain, G. A.; Berry, J. F. *J. Chem. Educ.* **2008**, *85*, 532–536.

EPR spectroscopy was performed on a Bruker EleXsys E-500 CW-EPR spectrometer. EPR samples were comprised of crystalline solid dissolved in butyronitrile to give a concentration of approximately 1.0 mM. EPR spectra were measured in frozen butyronitrile at non-saturating microwave powers of 0.6325 to 2 mW. EPR simulations were performed using EasySpin 5.0.2 as implemented in MATLAB.[6]

Spectral UV-vis measurements were recorded in THF or THF-$d_8$ on a Varian Cary 5000 spectrometer.

Cyclic voltammetry (CV) measurements were performed using a CH Instruments 730C potentiostat using a freshly polished glassy carbon working electrode (area = 0.07 $cm^2$), a platinum wire auxiliary electrode, and a silver wire reference electrode in THF (0.2 M [TBA][$PF_6$]) separated from the working solution by a Vycor frit. At the conclusion of each CV experiment, ferrocene was added, and all potentials were referenced to the ferrocenium/ferrocene ($Fc^{+/0}$) couple.

Mass spectrometry was performed on an Agilent 6220 ESI-TOF spectrometer in anionic mode. Samples were prepared in THF in a nitrogen-filled glovebox and placed into a 1.5 mL GC vial with a septum to prevent exposure to moisture and oxygen and immediately analyzed by ESI mass spectrometry.

### X-ray crystallographic details

Low-temperature diffraction data for single crystals were collected on a three-circle diffractometer coupled to a Bruker-AXS Smart Apex charged-coupled-device (detector) with graphite-monochromated Mo Kα radiation ($\lambda$ = 0.71073 Å) performing $\varphi$ and $\omega$ scans. The data were integrated using SAINT (Bruker AXS) and scaled with a multiscan absorption correction using SADABS (Bruker AXS).[7] The structures were solved by direct methods using SHELXS and refined against $F^2$ on all data by full-matrix least squares with SHELXL-97 using established methods.[8] Hydrogen atoms were placed in calculated positions using the standard riding model and refined isotropically. All non-hydrogen atoms were refined anisotropically.[9] In the structure of **1**, one of the ditox ligands is disordered over two positions, and each THF molecule of **1** is disordered over two positions; all disorder is satisfactorily modelled. In the structure of **3**, one of the 15-crown-5-ether rings was satisfactorily modelled over two positions.

### SQUID magnetometry details

SQUID magnetometry was carried out using a Quantum Design MPMS-5S SQUID

[6] Stoll, S.; Schweiger, A. *J. Magn. Reson. 178*, **2006**, 42–55.

[7] *Apex II*. Bruker AXS: Madison, WI, 2009.

[8] Sheldrick, G. M. *Acta Crystallogr., Sect. A: Found. Crystallogr.* **2008**, *64*, 112–122.

[9] Müller, P. *Crystallogr. Rev.* **2009**, *15*, 57–83.

magnetometer. Samples for SQUID were prepared as follows: free-flowing powder samples of **3** (ca. 30 mg) and eicosane wax (ca. 10 mg) were packed into a polycarbonate capsule under a dry nitrogen atmosphere. Warm liquid eicosane was added to suspend the material in solid wax after cooling. DC susceptibility measurements were collected in the temperature range of 2–298 K in 4 K increments under a DC field of 10 000 Oe. DC magnetization measurements were obtained in the temperature range of 2–298 K, under DC fields of 10, 20, 30, 40, 50, 60, and 70 kOe. The data collected were corrected for any diamagnetic contributions by comparison to an eicosane/capsule blank and by applying Pascal's constants to account for any core diamagnetism.[2]

### Syntheses and characterization of compounds

**Synthesis of HDitox.** To an oven dried 500 mL Schlenk flask charged with a teflon stirbar was added hexamethylacetone (10.0 g, 70.3 mmol). The reaction was cooled to 0 °C in an ice bath and methyllithiium (1.6 M in $Et_2O$) (50.0 mL, 80.0 mmol) was added slowly over the course of 20 min. The resulting solution was allowed to stir at 0 °C for an additional 30 min and then warmed to room temperature. The solvent was removed in vacuo and water was added. The reaction was then extracted with 3 × 50 mL of pentane, the organic layers collected, dried over sodium sulfate and the solvent removed at a temperature not exceeding 30 °C. The resulting pasty solid was dried under vacuum briefly with a secondary trap to yield H(ditox) as a bright white powder (9.57 g, 60.5 mmol, 86.1% yield).

**Synthesis of $Mn^{II}(ditox)_3K(THF)_2$ (1).** $MnCl_2$ (200 mg, 1.59 mmol) was combined with K(ditox) (936 mg, 4.77 mmol), and THF (10 mL) was added at room temperature. The resultant faintly yellow mixture was stirred at room temperature for 3 h, brought to dryness *in vacuo*, and pumped *in vacuo* at room temperature overnight. Complex **1** was then extracted with 3 × 10 mL portions of pentane, which were passed through a glass fiber filter. The pale yellow filtrate was concentrated to minimal volume and cooled at –40 °C overnight. Colorless crystals of complex **1** were grown, collected, and dried *in vacuo* (54.3% yield, 614 mg, 0.865 mmol). Anal. Calc'd. (Found) for $C_{38}H_{79}KO_5Mn$: C, 64.26 (63.97); H, 11.23 (10.94). IR ($cm^{-1}$): 1485 (w), 1476 (w), 1395 (w), 1381 (m), 1369 (w), 1358 (w), 1293 (w), 1249 (w), 1188 (w), 1136 (m), 1113 (s), 1094 (s), 1053 (s), 1021 (w), 1013 (w), 1001 (s), 930 (m), 908 (s), 864 (m), 763 (w), 659 (w), 637 (w), 575 (s), 555 (w), 535 (w), 453 (m). No discernible features in the UV-vis spectrum. $\mu_{eff}$ (Evans method) = 6.03 $\mu_B$. The X-ray crystal structure of **1** is provided in Figures 2 and S2.

**Preparation of $[Mn^{II}(ditox)_3][K(15\text{–crown–}5\text{–ether})_2]$ (2).** A solution of **1** (1.00 g, 1.41 mmol) in 10 mL of pentane was treated with 15–crown–5–ether (620 mg, 2.82 mmol) at room temperature and immediately gave a colorless precipitate. The reaction mixture was stirred as a colorless slurry for 30 min, after which time stirring was ceased and a colorless solid settled to the bottom of the reaction vessel. The supernatant was removed via pipette. The solid was washed with 3 × 10 mL portions of pentane. The solid was then pumped *in*

*vacuo* at room temperature for 1 h to give **2** as a fine colorless powder (94.4 % yield, 1.43 g, 1.33 mmol). The solid was dissolved in a minimal amount of $Et_2O$ and chilled at –40 °C overnight to give **2** as colorless crystals (31.1 % yield, 442 mg, 0.439 mmol). Anal. Calc'd. (Found) for $C_{50}H_{103}KO_{13}Mn$: C, 59.66 (59.33); H, 10.34 (10.39). IR ($cm^{-1}$): 1603(w), 1475 (w), 1445 (w), 1401 (w), 1375 (w), 1354 (m), 1303 (w), 1289 (w), 1253 (w), 1119 (s), 1090 (w), 1039 (w), 1008 (w), 940 (m), 915 (w), 855 (m), 830 (w), 764 (w), 646 (w), 583 (w), 551 (w), 519 (w), 508 (w). No discernible features in the UV-vis spectrum. $\mu_{eff}$ (Evans method) = 5.77 $\mu_B$. The X-ray crystal structure of **2** is provided in Figures 2 and S3.

**Preparation of [$Mn^{IV}$(O)(ditox)$_3$][K(15–crown–5–ether)$_2$] (3).** The following manipulations were performed in the dark. The complex **2** (1.45 g, 1.44 mmol) was combined with PhIO (317 mg, 1.44 mmol), and THF (10 mL) was added at room temperature. The mixture was stirred for 1 h at room temperature. A dark green mixture resulted within minutes. After 1 h, the mixture was brought passed through a glass fiber filter, and the dark green filtrate was brought to dryness *in vacuo*. The resultant dark green solid was washed with 3 × 10 mL portions of pentane and then pumped *in vacuo* at room temperature for 1 h to give a fine free-flowing powder (79.3 % yield, 1.15 g, 1.12 mmol). Anal. Calc'd. (Found) for $C_{50}H_{103}KO_{14}Mn$: C, 58.72 (58.57); H, 10.17 (9.98). IR ($cm^{-1}$): 1586 (w), 1475 (w), 1445 (w), 1394 (w), 1382 (w), 1354 (m), 1304 (w), 1289 (w), 1253 (w), 1245 (w), 1193 (w), 1120 (s), 1105 (s), 1091 (s), 1040 (m), 1032 (m), 999 (s), 940 (s), 905 (s), 855 (s), 846 (s), 830 (m), 765 (w), 648 (s), 590 (s), 552 (w), 519 (w), 508 (w). UV-vis-NIR (THF): λ/nm (ε/$M^{-1}$ $cm^{-1}$) 523 (250), 583 (230), 832 (52.9), 941 (63.5). $\mu_{eff}$ (298 K, Evans method) = 4.54 $\mu_B$. $\mu_{eff}$ (298 K, SQUID) = 4.75 $\mu_B$. ESI-MS$^-$ (*m/z*) 542.4 [Mn(O)(ditox)$_3$]$^-$. Dark green X-ray quality crystals were obtained from a saturated $Et_2O$ solution chilled at –40 °C overnight. The X-ray crystal structure of **3** is provided in Figures 2 and S4.

(a)

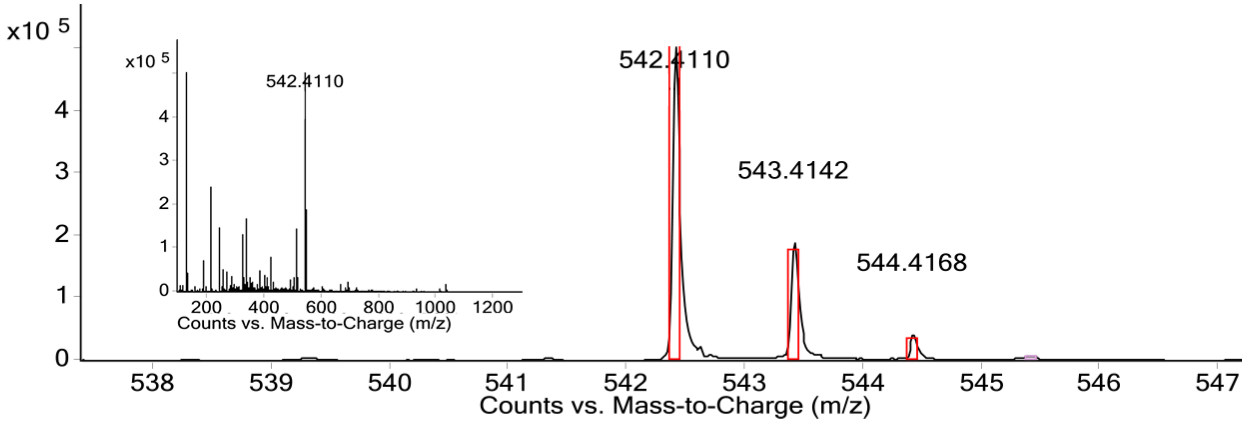


| Observed (*m/z*) | Calculated (*m/z*) | *z* | Relative Abundance | Formula |
|---|---|---|---|---|
| 542.4110 | 542.4112 | $1^-$ | 12.0 | $[C_{30}H_{63}MnO_4]^-$ |
| 543.4142 | 543.4147 | $1^-$ | 4.53 | $[C_{30}H_{63}MnO_4]^-$ |
| 544.4168 | 544.4177 | $1^-$ | 1.00 | $[C_{30}H_{63}MnO_4]^-$ |

[a] negative ion mode

(b)

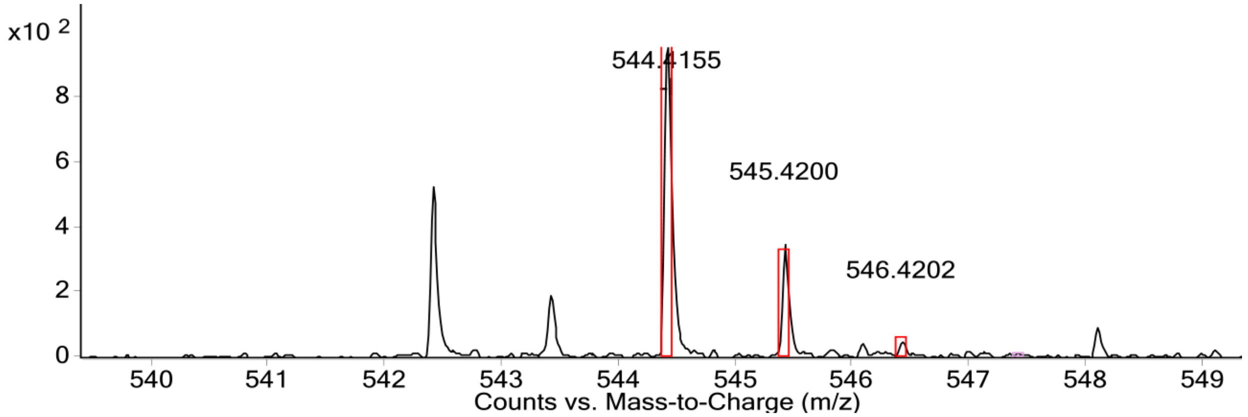


**Figure S1**. (a) ESI-MS spectrum of $[Mn^{IV}(O)(ditox)_3][K(15\text{-crown-5-ether})_2]$ (**3**) in THF and a table of data pertaining to the observed and predicted isotopic distribution pattern. (b) ESI-MS spectrum of $^{18}O_{oxo}$-enriched $[Mn^{IV}(O)(ditox)_3][K(15\text{-crown-5-ether})_2]$ (**3**).

**Table S1**. Crystal data and structure refinement for **1-3**

| | **1** | **2** | **3** |
|---|---|---|---|
| formula | $C_{38}H_{79}KMnO_5$ | $C_{50}H_{103}KMnO_{13}$ | $C_{50}H_{103}KMnO_{14}$ |
| Fw, g/mol | 710.05 | 1006.36 | 1022.36 |
| temperature | 100(2) K | 100(2) K | 100(2) K |
| cryst system | monoclinic | monoclinic | triclinic |
| space group | *C*2/c | $P2_1$/n | *P*-1 |
| color | colorless | colorless | green |
| *Z* | 4 | 4 | 2 |
| *a*, Å | 18.3234(11) | 11.3584(5) | 11.5253(8) |
| *b*, Å | 15.9361(10) | 17.2479(8) | 16.1662(11) |
| *c*, Å | 16.7710(11) | 29.1110(13) | 16.7311(12) |
| *α*, deg | 90.00 | 90.00 | 81.9430(10) |
| *β*, deg | 122.093(1) | 91.5520(9) | 88.4630(10) |
| *γ*, deg | 90.00 | 90.00 | 69.6350(10) |
| *V*, $Å^3$ | 4148.8(5) | 5701.0(4) | 2892.8(3) |
| $d_{calc}$, $g/cm^3$ | 1.137 | 1.173 | 1.174 |
| *μ*, $mm^{-1}$ | 0.455 | 0.360 | 0.357 |
| 2*θ*, deg | 55.86 | 52.74 | 52.74 |
| $R1^a$ (all data) | 0.0611 | 0.1180 | 0.0939 |
| $wR2^b$ (all data) | 0.1447 | 0.2413 | 0.1088 |
| $R1^b$ [(I>2σ)] | 0.0518 | 0.0830 | 0.0489 |
| *wR*2 [(I>2σ)] | 0.1354 | 0.2151 | 0.0942 |
| GOF ($F^2$) | 1.032 | 1.043 | 1.004 |

$^aR1 = \Sigma||F_o - |F_c||/\Sigma|F_o|$. $^b wR2 = (\Sigma(w(F_o^2 - F_c^2)^2)/\Sigma(w(F_o^2)^2))^{1/2}$. $^c GOF = (\Sigma w(F_o^2 - F_c^2)^2/(n - p))^{1/2}$ where *n* is the number of data and *p* is the number of parameters refined.

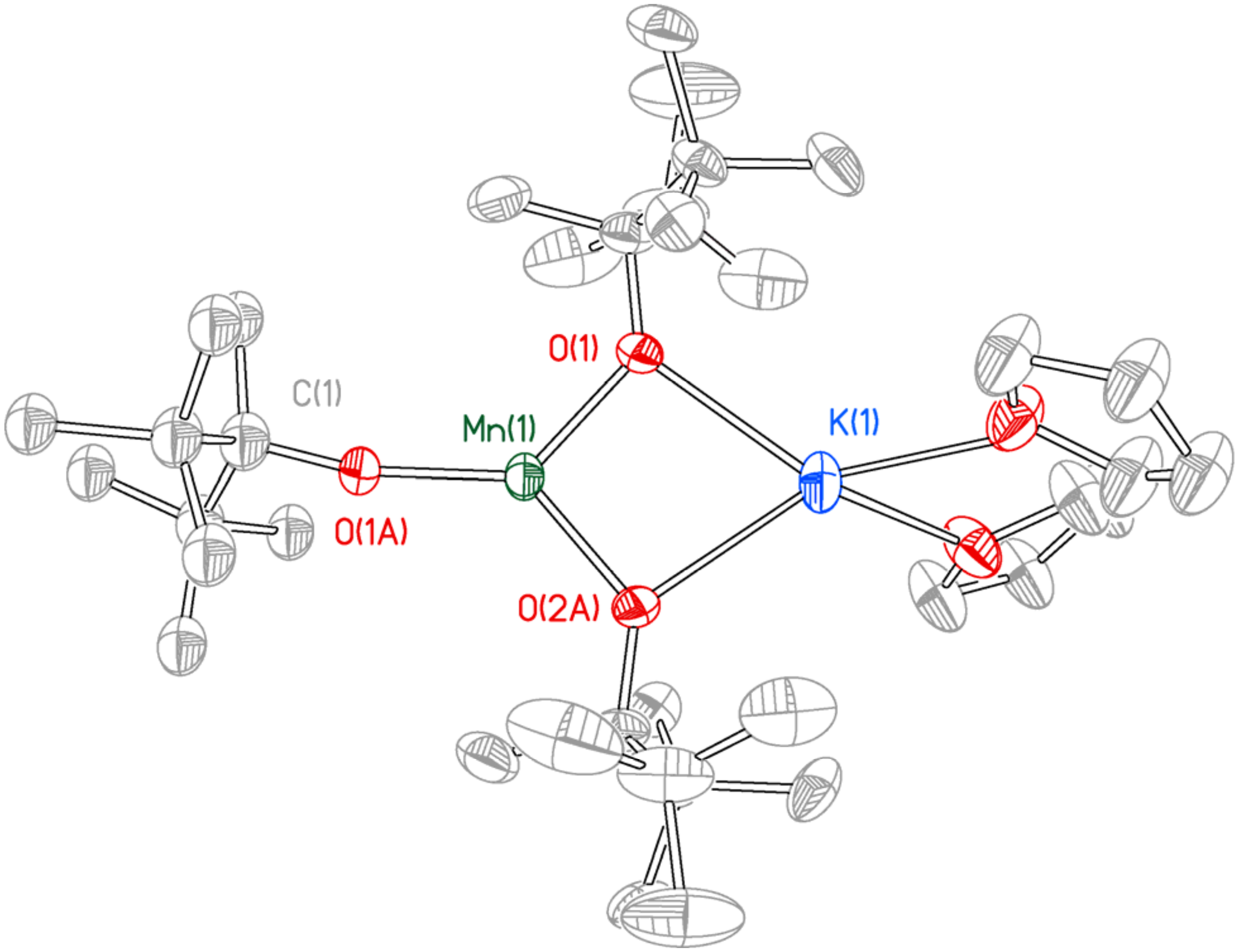


**Figure S2.** Structure of Mn(ditox)$_3$K(THF)$_2$ (**1**), 50% probability ellipsoids. Hydrogen atoms omitted for clarity. Selected bond distances: *d*(Mn1–O1) = 1.971(1) Å, *d*(Mn1–O2A) = 1.881(3) Å, *d*(K1–O1) = 1.971(1) Å. Selected bond angles: ∠(O1–Mn1–O2A) = 131.7(2)°, ∠(K1–O1–Mn1) = 95.8(1)°, ∠(O1–Mn1–O1A) = 97.6(1)°.

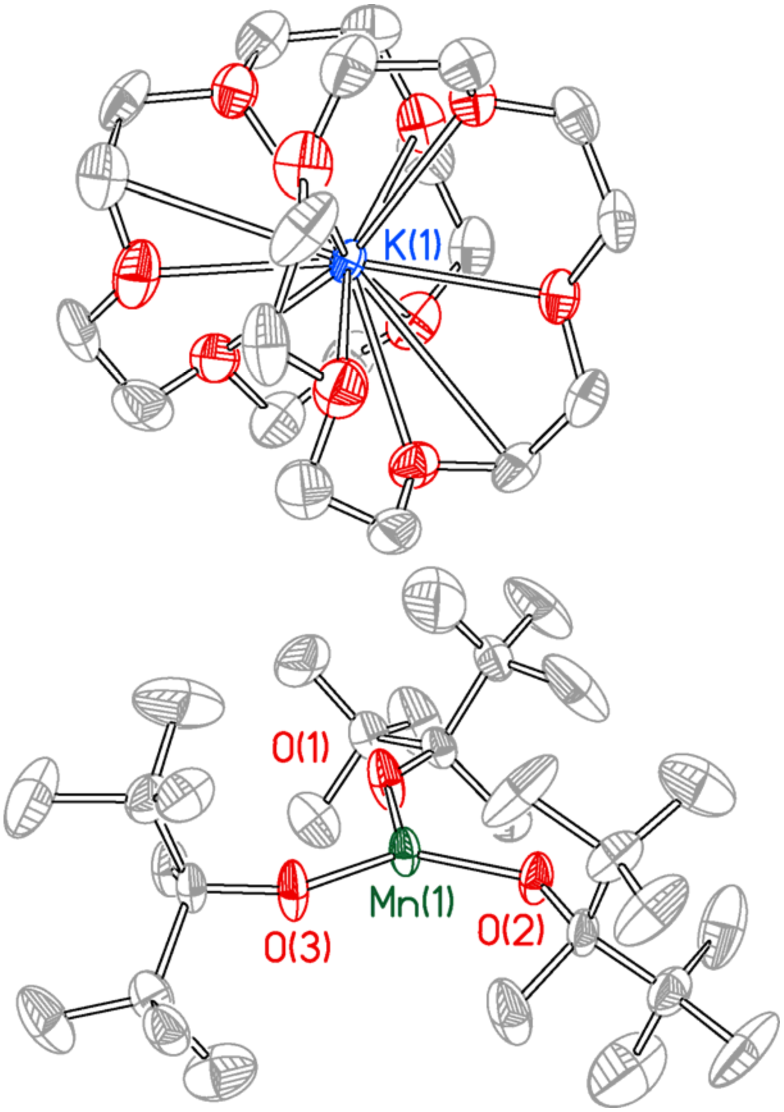


**Figure S3.** Structure of $[Mn^{II}(ditox)_3][K(15\text{-crown-5-ether})_2]$ (**2**), 50% probability ellipsoids. Hydrogen atoms omitted for clarity. Selected bond distances: *d*(Mn1–O1) = 1.925(4) Å, *d*(Mn1–O2) = 1.931(4) Å, *d*(Mn1–O3) = 1.929(4) Å. Selected bond angles: ∠(O1–Mn1–O2) = 120.0(2)°, ∠(O2–Mn1–O3) = 120.2(2)°, ∠(O3–Mn1–O1) = 119.6(2)°.

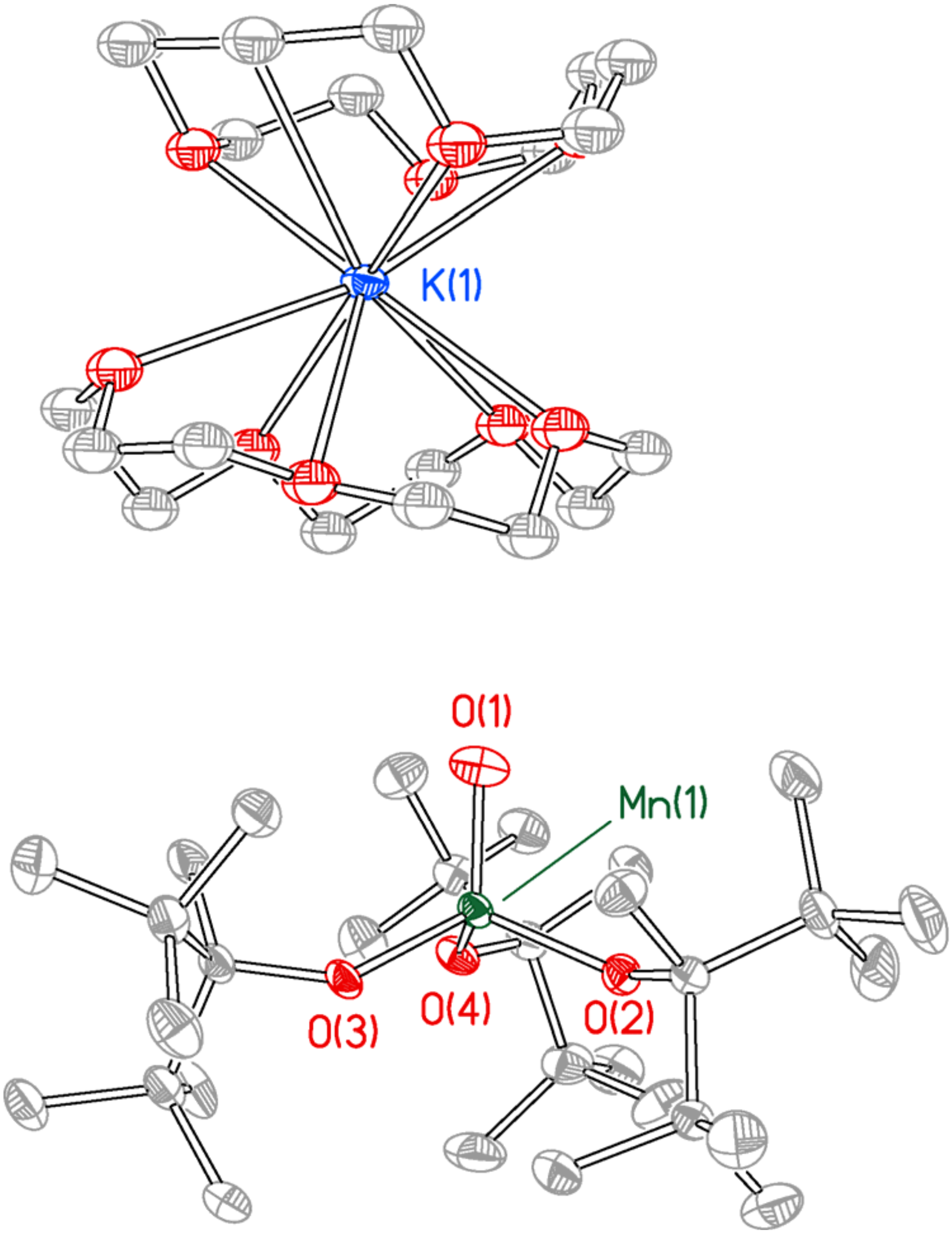


**Figure S4.** Structure of [$Mn^{IV}$(O)(ditox)$_3$][K(15-crown-5-ether)$_2$] (**3**), 50% probability ellipsoids. Hydrogen atoms omitted for clarity. Selected bond distances: *d*(Mn1–O1) = 1.628(2) Å, *d*(Mn1–O2) = 1.876(2) Å, *d*(Mn1–O3) = 1.843(1) Å, *d*(Mn1–O4) = 1.828(2) Å. Selected bond angles: ∠(O1–Mn1–O2) = 107.08(8)°, ∠(O1–Mn1–O3) = 110.57(8)°, ∠(O1–Mn1–O4) = 116.04(8)°, ∠(O2–Mn1–O3) = 110.78(7)°, ∠(O2–Mn1–O4) = 108.73(7)°, ∠(O3–Mn1–O4) = 103.62(7)°.

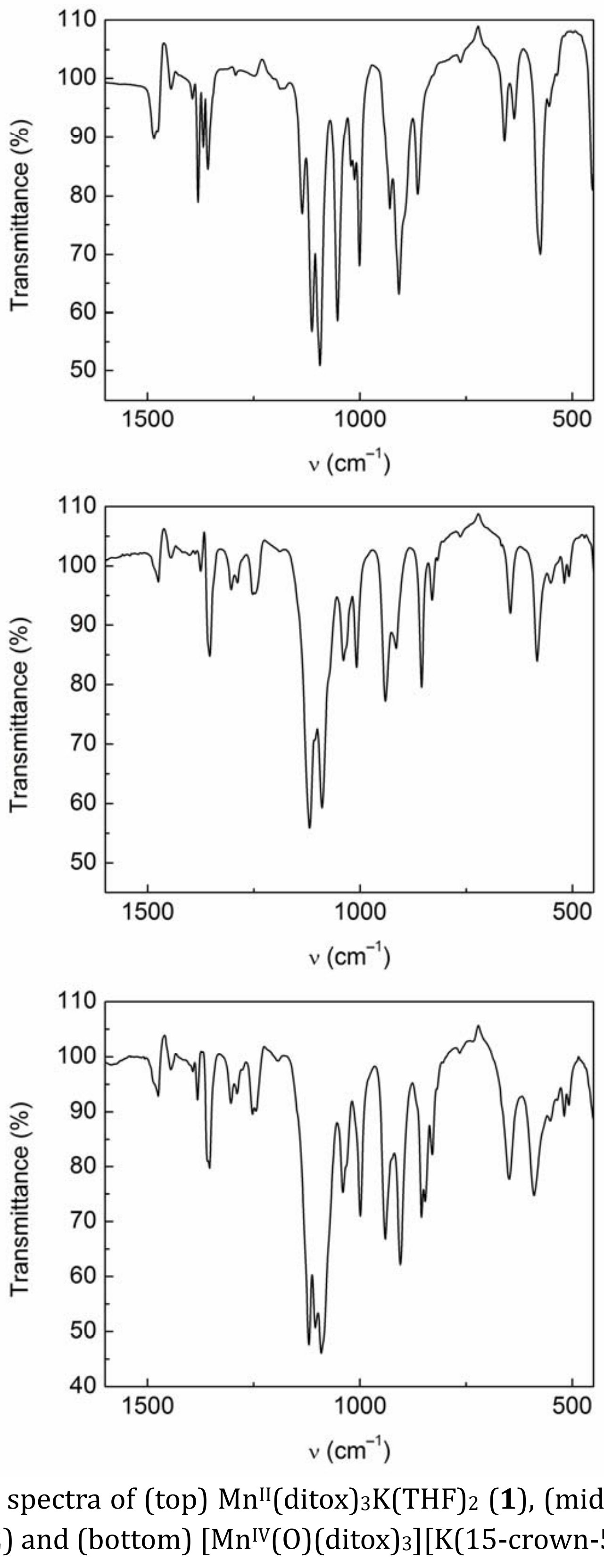


**Figure S5**. Infrared spectra of (top) $Mn^{II}(ditox)_3K(THF)_2$ (**1**), (middle) $[Mn^{II}(ditox)_3][K(15\text{-crown-5-ether})_2]$ (**2**) and (bottom) $[Mn^{IV}(O)(ditox)_3][K(15\text{-crown-5-ether})_2]$ (**3**).

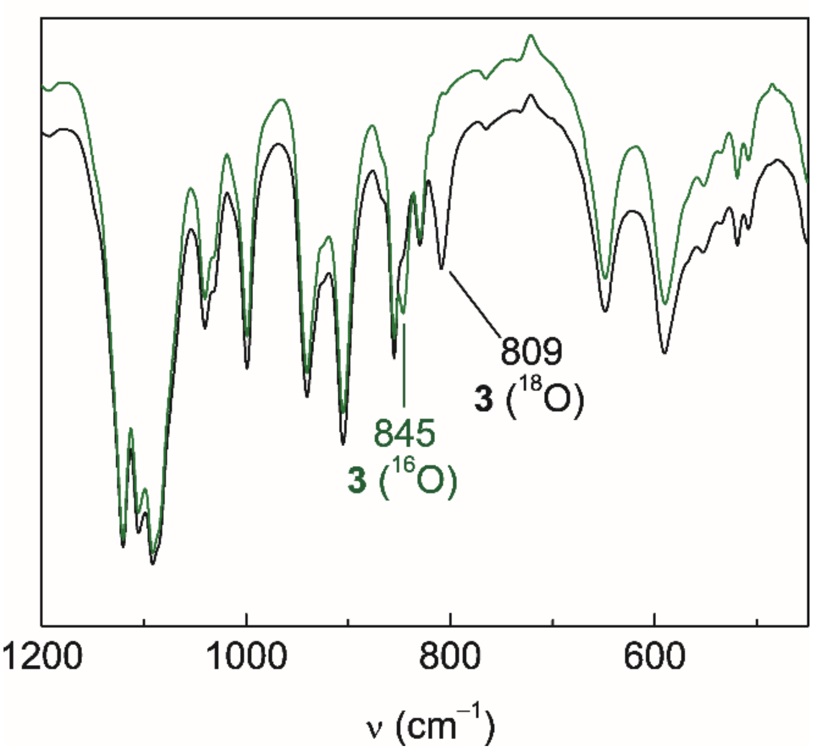


**Figure 6.** Comparison of the IR spectra in the 1200-450 $cm^{-1}$ region for the different isotopomers of $[Mn^{IV}(O)(ditox)_3][K(15\text{-}C\text{-}5)_2]$ (**3**). Numbers indicate stretching frequency for the Mn-oxo for Mn–$^{18}O$ (black, ▬) and Mn–$^{16}O$ (green line, ▬).

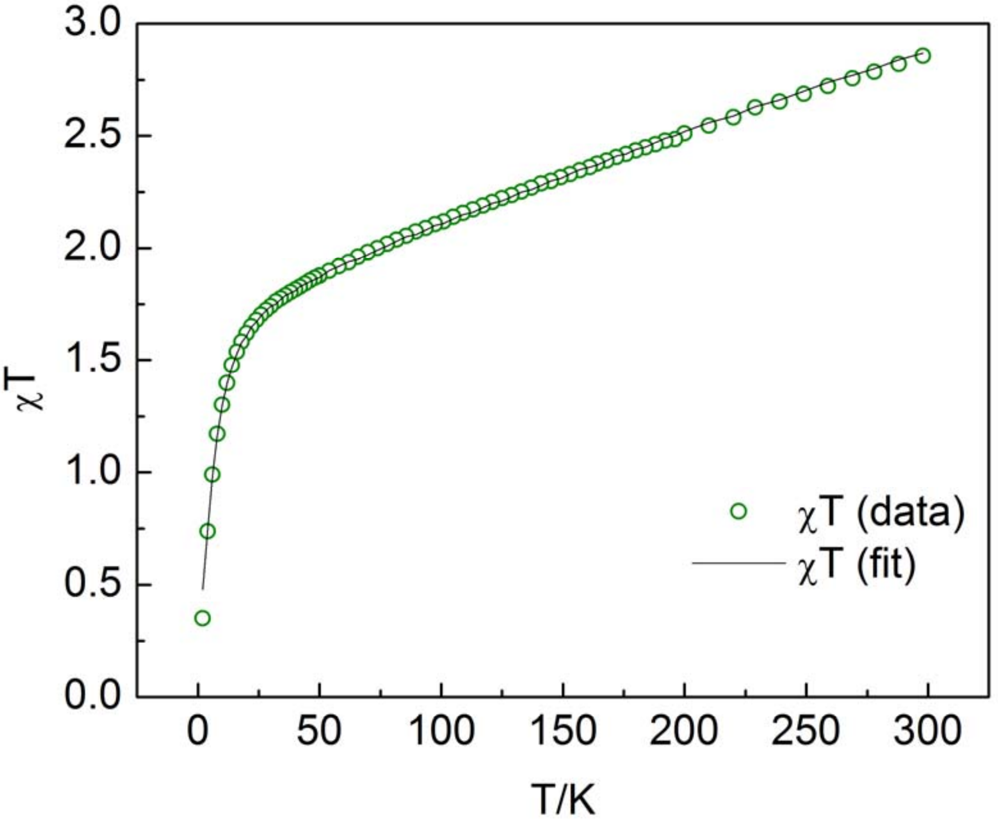


**Figure S7**. SQUID data $[Mn^{IV}(O)(ditox)_3][K(15\text{-crown-5-ether})_2]$ (**3**). The black line is a simulation with D = –12.31 cm$^{-1}$ $g_x$ = 4.15, $g_y$ = 3.57, $g_z$ = 1.92.

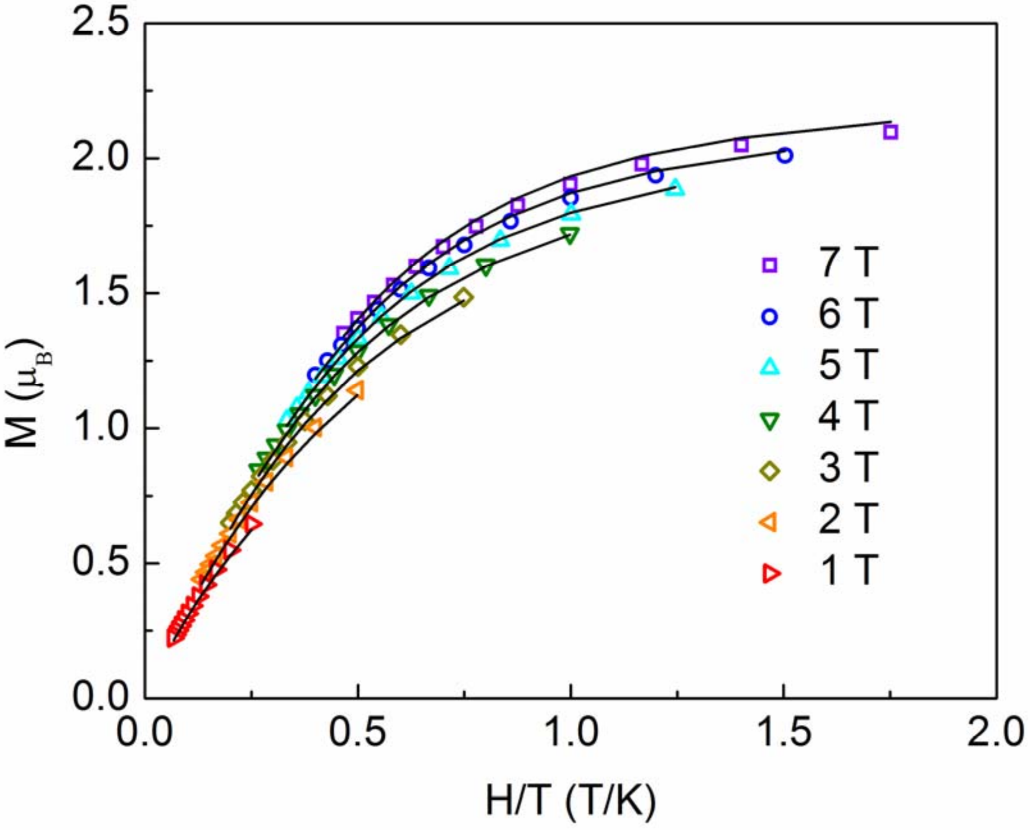


**Figure S8.** SQUID reduced magnetization data [$Mn^{IV}$(O)(ditox)$_3$][K(15-crown-5-ether)$_2$] (**3**). Black lines are simulations with D = –23.4 cm$^{-1}$, |E| = 6, $g_{avg}$ = 3.13.

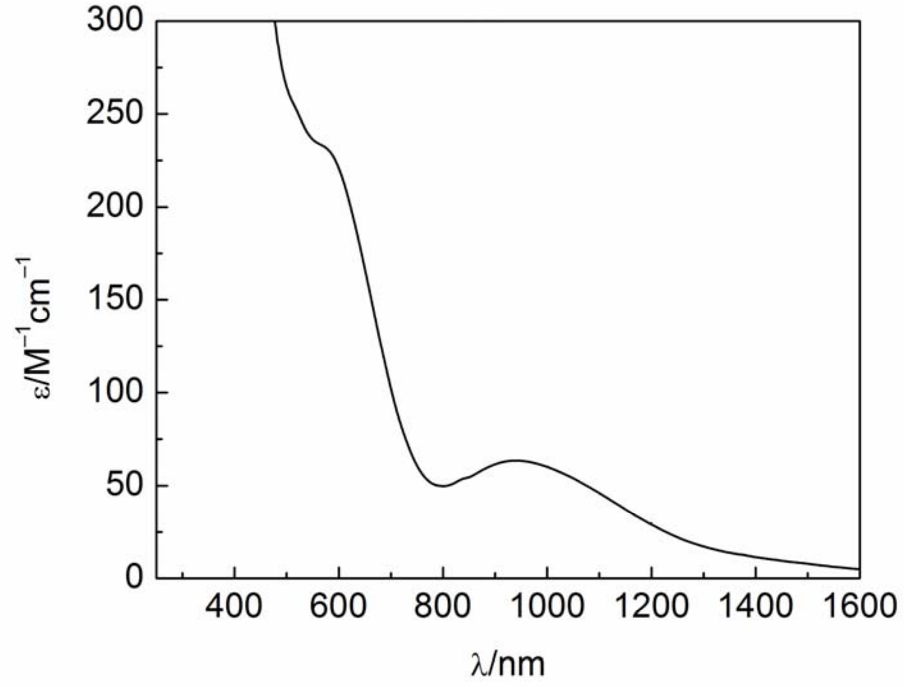


**Figure S9**. UV-vis spectrum of $[Mn^{IV}(O)(ditox)_3][K(15\text{-crown-5-ether})_2]$ (**3**) in THF-$d_8$.

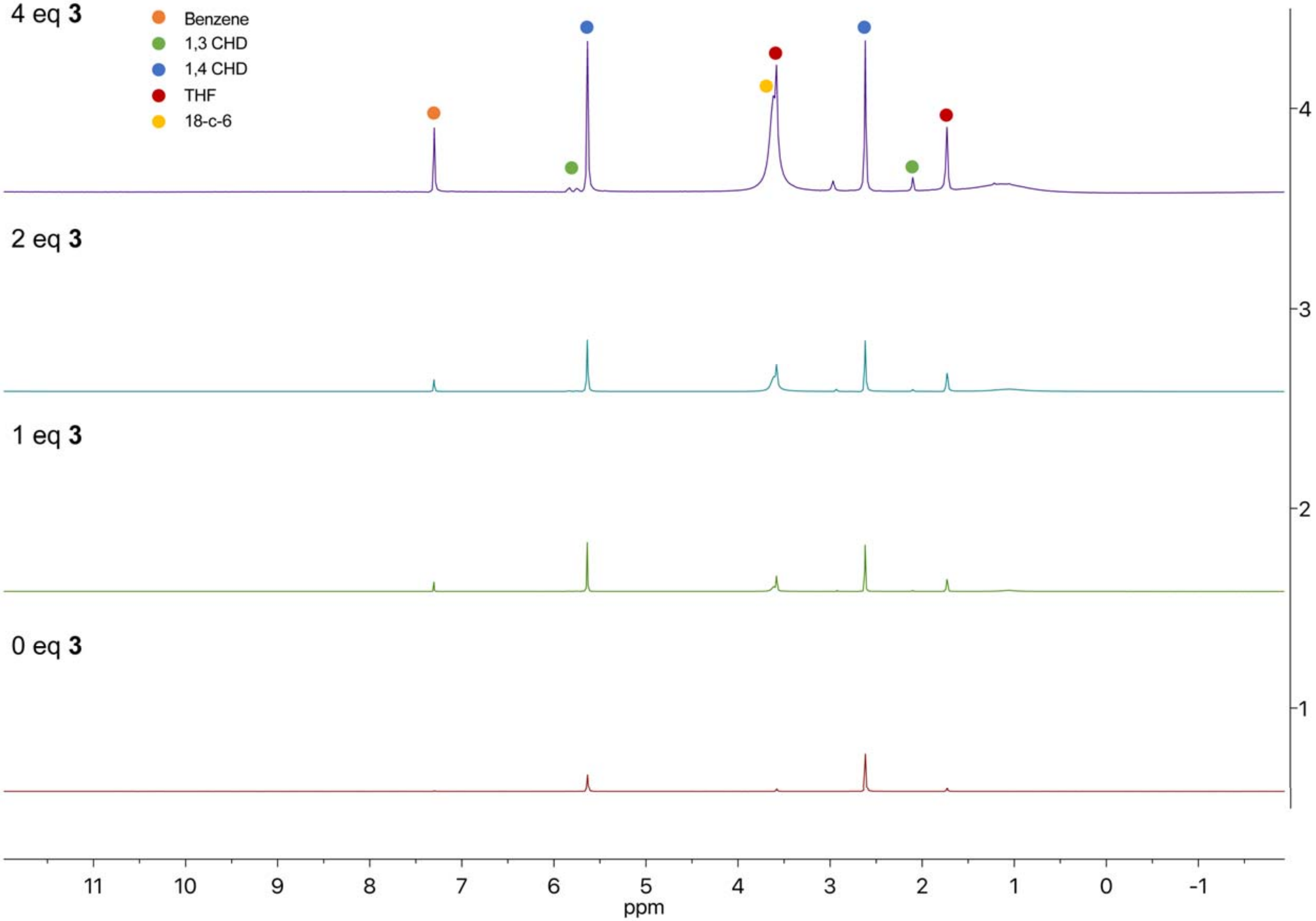


**Figure S10.** $^{1}H$ NMR showing the reaction of **3** with increasing number of equivalents of 1,4-cyclohexadiene (CHD).
